# Incorporating Virtues: A Speech Act Approach to Understanding how Virtues Can Work in Business

**DOI:** 10.1007/s40926-021-00171-3

**Published:** 2021-03-10

**Authors:** Todd Mei

**Affiliations:** Department of Philosophy, University of Kent, Canterbury, CT2 7NF UK

**Keywords:** Business virtue ethics, Speech act theory, Perlocution, MacIntyre, Ricoeur

## Abstract

One of the key debates about applying virtue ethics to business is whether or not the aims and values of a business actually prevent the exercise of virtues. Some of the more interesting disagreement in this debate has arisen amongst proponents of virtue ethics. This article analyzes the central issues of this debate in order to advance an alternative way of thinking about how a business can be a form of virtuous practice. Instead of relying on the paired concepts of internal and external goods that define what counts as virtuous, I offer a version of speech act theory taken from Paul Ricoeur to show how a business can satisfy several aims without compromising the exercise of the virtues. I refer to this as a polyvalent approach where a single task within a business can have instrumental, conventional, and imaginative effects. These effects correspond to the locutionary, illocutionary, and perlocutionary dimensions of meaning. I argue that perlocution provides a way in which the moral imagination can discover the moral significance of others that might have not been noticed before, and furthermore, that for such effects to be practiced, they require appropriate virtues. I look at two cases taken from consultation work to thresh out the theoretical and practical detail.

The agent-focused, or person-oriented, approach of virtue ethics can be a welcome fit for businesses seeking to provide a greater sense of professional and personal development for their employees. This is mainly due to the way virtues are character traits or intellectual features that help determine how we might act in view of some greater end or goal. Virtues can therefore complement corporate values that have a more over-arching intent to structure how employees act. But virtues are not just aspects of a person’s make-up; there can also be corporate-focused virtues that a business might decide its employees ought to cultivate.

A particularly good resource for this kind of application of virtues is Alasdair MacIntyre’s account of virtuous practice since it can arguably help businesses understand in what ways their tasks and operations can be profitable as well as morally responsible. The most prominent advocate of applying MacIntyre’s ideas in this way is Geoff Moore (Moore 2017, 2015, 2008, 2005, 2002; cf. Moore and Beadle 2006, Horvath 1995). However, Moore is not without his critics; and surprisingly, the most pronounced of these criticisms comes from proponents of MacIntyre’s moral philosophy who allege that the way MacIntyre conceives practices and moral goods actually prohibits an application to business. I will later describe this point of contention in terms of an *either/or* dualism, where the internal goods arising from virtuous action are either uncontaminated by the external goods of business, such as profit, or they are not.

The aim of this article is not to resolve the debate between Moore and his critics, but to use its discussion to set up an alternative approach using a version of speech act theory to understand how business can be a form of moral practice, and subsequently, how virtues are integral to the cultivation of this practice.

Key to my approach is Paul Ricoeur’s (1973) philosophy of action, wherein he reverses the direction typical of speech act theory by demonstrating how action bears the linguistic features of locution, illocution, and perlocution. A general advantage of Ricoeur’s theory is that it delineates how action predicates meanings in different ways. A single action can articulate, express, or perform several meanings at once. When applied to the realm of business, it will become apparent how any task performed by an employee has different levels of meaning that constitute a richer landscape in which an employee can evaluate her actions with regard to respective ends.

I will refer to this way of using speech act theory as a *polyvalent approach*, as opposed to MacIntyre’s dualistic one. Valences refer to the business organizational ends that a *single* action can predicate. As we will see, the action an employee performs can be understood as meeting ends relating to virtuousness as well as business success.

The one caveat of the polyvalent approach is that in comparison to MacIntyre’s distinction about internal and external goods, it seems to lack a measure by which one can determine when an action that appears virtuous on the surface is actually done for the wrong ends. If an action has multiple valences, then it seems one can easily be uncritical or indiscriminate as to the effects of what has been achieved. One can simply emphasize a valence that is taken to be good over another valence that is questionable or even morally harmful. I will refer to this problem as “MacIntyre’s objection”, and though I will provide a response later, it is worthwhile making some anticipatory remarks.

To illustrate both how polyvalence works and how it does not fall prey to MacIntyre’s objection, I will draw on Ricoeur’s distinct emphasis on the importance of perlocution. Perlocution is often not the focus of conventional speech act analysis (Mei 2019a). But on Ricoeur’s view, perlocution should not be overlooked because it tends to involve imaginative effects that have the potential to transform the way we think and see things. Imaginative effects, as we will see, have the ability to expand and critically revise our moral thinking. In short, perlocution offers both the means to moral awareness as well as a capacity to reflect critically on how one’s work and business square with this awareness.

My analysis is divided into five sections. Section one examines MacIntyre’s account of virtuous practice and the debate surrounding its application to business. Section two advances a speech act theoretical account of how moral recognition can arise in the everyday transactions of businesses. Section three extends this argument by showing how virtues are integral to the development of this moral recognition. Section four responds to MacIntyre’s objection. Section five concludes with speculation on the practical implications of the speech act approach.

## MacIntyre’s either/or: Internal V. External Goods

By a practice I am going to mean any coherent and complex form of socially established cooperative human activity through which goods internal to that form of activity are realized in the course of trying to achieve those standards of excellence which are appropriate to, and partially definitive of, that form of activity, with the result that human powers to achieve excellence, and human conceptions of the ends and goods involved, are systematically extended.

MacIntyre (2007, 218)

MacIntyre’s definition of virtuous practice is often cited since, as we will see, the bar for what counts as a deliberative, virtuous activity is set quite high. This definition takes seriously the idea that being morally good should not simply fall to following a set of rules or invoking measurements of consequences that one might count as benevolent. Being morally good should entail a kind of activity where the activity itself is informed by and further forms our moral reasoning and character. Key to this account is how MacIntyre distinguishes between internal and external goods.

When practicing a virtue one accomplishes something specific to that virtue that is good in itself and is not good for some other end. Other ends may come about, but they are by-products of the action. So, for example, in practicing temperance one accomplishes being temperate in that situation. One can say such an accomplishment is internal to the practice of that virtue and that being temperate is a good in itself; one is not temperate for the sake of any other good. Aristotle understood this in terms of the immediacy of ethical action which, in exercising a virtue, involves an identity where the performance of the action is also the achievement of the end of the action.^1^ If it so happens that being temperate also results in someone praising you for moderation, then that would count as an external good that is contingent to what it means to be temperate. Being temperate is its own end, and not done for the end of receiving praise.

MacIntyre’s (2007) critical supplement is to examine how virtues necessarily involve practices. One cannot just be virtuous in the abstract; virtues need to be exercised according to what it means to do something virtuously in some particular context. There is also an important epistemological requirement to this that I will discuss later. For now, let us return to our example to see why one cannot be virtuous in the abstract. Being temperate can only really make sense when it is set within certain norms of why one ought to do something temperately. These norms tend to indicate standards of excellence, and these standards define how temperance is to be exercised for that context. It is therefore the exercise of virtues within this context of excellence that define their practice. Moreover, when doing so the exercise of virtues ideally results in the achievement of goods internal to that practice. So to elaborate our example in terms of providing context, let us take temperance as it is exercised in reasoned debate. One can say that temperance is one of the virtues necessary for the practice of reasoned debate, not only in terms of politeness to one’s interlocutor but also as a way to preserve an openness of mind when hearing opposing views. The internal goods of debate that might be achieved by being temperate are the articulation, validation, and recognition of truth. Indeed, these are the goods that we take to be “appropriate to, and partially definitive of, that form of activity” (MacIntyre 2007, 229–230) we call debate.

It is clear on this account that internal goods are what matter to being virtuous; otherwise there would be no sense in doing something virtuously. A debate won by vicious means—for example, by intellectual bullying—does not meet those standards of excellence we expect of reasoned debate and the ends associated with the process of analysis and argumentation.

However, we should not forget that the Aristotelian-influenced form of virtue ethics allows for us to take pleasure in goods external to a moral practice. Praise, monetary reward, publicity. . . such goods can be enjoyed but they are not to be mistaken as the ends of virtuous action. Thus, we should never be deceived into thinking that we are virtuous in order to gain these kinds of goods. When this deception does occur, let us call it *an error in the ordering of goods*. This error is precisely why the idea of business as a moral practice is at the very least problematic, if not impossible. Within the context of business, it is MacIntyre’s worry that the ordering is compromised, such that the external goods of profit govern how the virtues are exercised and are understood.

What if a business trains its employees to practice temperance for the end of obtaining more clients (cf. Dewar et al. 2019)? Has temperance been called into service by some other end, such as growth or profit? If that is the case, then the internal goods integral to a virtuous practice have been replaced by an external good. MacIntyre notes:

[A]lthough we may hope that we can not only achieve the standards of excellence and the internal goods of certain practices by possessing the virtues *and* becoming rich, famous, and powerful, the virtues are always a potential stumbling block to this comfortable ambition.

MacIntyre (2007, 228)

More recently:

Workplaces by contrast may be organized so that the work performed by individuals is never more than a cost-effective means to ends imposed by others for the sake of high productivity and profitability.

MacIntyre (2016, 172)^2^

Businesses sit within the zone of comfortable ambition because one can easily be enthralled by those ends that cater to success and also require less self-critical scrutiny about the conduct of one’s life. So it is not only the case that such external goods can interfere with an understanding of virtue, but it is also the worry that in utilizing virtues and being driven ultimately by profit, the virtues will be instrumentalized for external goods. Virtues lose their relation to internal goods, and we understand them merely as the means to some other end. This is precisely the criticism put by Ron Beadle (2008) and Matthew Sinnicks (2014) when discussing Moore’s (2002, 2005) attempt to use aspects of MacIntyre’s philosophy to describe business as virtuous practice. Simply put, MacIntyre’s moral philosophy cannot *conceptually accommodate* the institution of business because its institution is defined by the kinds of aims and ambitions that block the virtues (cf. Beadle and Knight 2012, 437).

Let us note an important implication of this conceptual feature in terms of the epistemological requirement that I mentioned earlier. That which is internally good is not only something innate to a practice but arises only insofar as the practitioner is aware of what the practice is aiming at and what virtues are necessary for executing the practice well. So, in other words, there is an epistemological requirement where one can be a practitioner only by knowing how certain virtues are key to the respective practice; and conversely, one can speak of virtues only by knowing how the practice they inform help one to undertake said practice. Sinnicks (2014, 4) refers to this as a “cognitively closed” conception of practice. The advantage of this account is that it sets the bar relatively high for anything to count as a practice. This feature also aligns with the typical virtue ethicist account that ethical action requires the exercise of one’s practical reasoning (*phronesis*) as opposed to following rules or procedures (Solomon 1992b, 175). In contrast, actions undertaken in the course of business tend not to have the same epistemological requisite for reflection; one merely needs to learn how to do certain tasks in view of ends like profit or efficiency. Practices, in other words, involve a substantial degree of critical self-reflection and deliberation (*phronesis*) whereas tasks in business can suffer a narrow focus on its immediate ends, or what is instrumental reasoning (*techne*) (cf. Gadamer 1981).

This last point about critical self-reflection cannot be underestimated. The either/or dualism in MacIntyre’s account is not a consequence of his distinction between internal and external goods. As we saw earlier, outside the context of business, the right ordered relation can be maintained where external goods are enjoyed as a by-product of ethical action and not as its end; and neither does the dualism results from MacIntyre’s worry that in business concerns for profit will more often than not compromise the exercise of the virtues. More precisely, what drives the dualism is the lack or compromise of one’s self-critical capacity. An employee may exercise a virtue, but she may be doing so for a non-virtuous end; and she most likely will not be able to notice this or not be compelled to do so. In other words, one can say that business tasks are also cognitively closed like virtuous practice. The difference is that by which business is enclosed happens to be concerns for goods like profit; and furthermore, these kinds of goods have no substantial conceptual link to moral concepts. Being good at business in no way entails being good morally. Business simply cannot count as a form of practice.^3^

It would be difficult to argue that this kind of conceptual closure does not accurately describe a key problem for business ethics (cf. Dempsey 2015). Yet, the worry is that on MacIntyre’s account business is precluded from the realm of ethics, and he provides no other way for us to classify business activity except as non-virtuous. This then results in a sort of determinism about business: Businesses cannot self-regulate since they lack the resources and disposition to do so. The best we can do is to employ some form of external regulation. Sinnicks therefore argues:

Regulation then is not simply a substitute for morality. When it is devised excellently, it can be a negative facilitator of morality even if it cannot directly foster a positive ethics (though we might more optimistically note that regulation could lead to certain habits which could, in due course, become virtues).

Sinnicks (2014, 243)

This strategy is somewhat problematic. Although regulation is no substitute for morality, it offers a regressive maneuver that assumes that an external authority is competent to diagnose and remedy problematic issues of corporate responsibility. We would then need to give some kind of account as to how this external authority is itself virtuous and not merely determining regulations and imposing regulations in a non-virtuous way.

But even if regulation was accepted as providing (negative) moral guidance, it is still arguable that it does not bring anything substantially ethical to business. So besides the problem about whether an external authority is itself ethical, another complication of Sinnick’s approach is that it assumes that an external authority can really institute change in such a way that it meets the epistemic requirement that is expected of moral practice (at least by virtue ethicists). If virtuous agents are those who deliberate and decide to do something for a significantly good reason, it is questionable if external sanction meets this criterion. As Ricoeur (Ricoeur 1992, 336–343; cf. Annas 2015) notes, to be told externally how to act is not the same as to come to a decision by oneself. Moral reasoning, per MacIntyre’s account of practice, requires the agent to understand what is good to do and why it is good to do it. So imposing regulation on business would at best only provide for a kind of behaviorism that mimics ethical action and cannot, as Sinnicks would like to suggest, “lead to certain habits which could, in due course, become virtues.” Habits can only become virtues if the agent in question has the critical reasoning capacity to see that such habits matter morally. For Sinnicks to admit this would mean that in some sense business is a practice.

Moore’s attempt to accommodate virtuous capacity within business organizations is therefore commendable since it grants that even businesses can be moral communities of some kind. But if the criticisms of Beadle and Sinnick are accurate, then the definitions of virtue and practice on which Moore relies are incompatible with his project. So instead of attempting to resolve this debate, we might seek an alternative way of describing business as a practice. What if the actions undertaken within business were all potentially candidates for virtuous action, not because they conform to the criteria MacIntyre sets out, but because they can form the kinds of connections we typically associate with moral awareness?

## The Moral Connection: From Goods to Speech Acts

Recalling what I mentioned earlier with respect to how Ricoeur takes action to bear the linguistic features of locutionary, illocutionary, and perlocutionary meanings, the alternative I propose is to see how the tasks employees undertake are polyvalent. (Due to the scope of this article, I will assume his theory is plausible.^4^) This approach to business will allow one to see a single action as capable of attaining different ends. In this sense, we avoid beginning with the supposition that external goods are by default competitors with virtue. Rather, we begin with the idea that action can fulfill business aims (i.e. external goods) as well as those that count as moral (i.e. internal goods). In writing a first-class essay, cannot a student satisfy the (internal) goods of arguing and analyzing well and achieving high marks and praise (external goods)?

The remit of this section concerns how the polyvalent approach accommodates business ends while being able to facilitate the recognition of a moral connection with others. Once we can see how polyvalence allows for this moral awareness, we can then turn to the role of virtues from a speech act theoretical point of view.

Let us consider that moral reasoning is predicated on the capacity to recognize a moral connection with others. This is because such a connection is a prerequisite to more substantial moral relations that one might, for example, describe under duties and virtues. To be sure, it is not merely the cognitive recognition that others exist, but the capacity to see others as having some kind of significant moral standing. Onora O’Neill puts this well:

By itself knowledge or belief that others exist will not bring them within the scope of ethical consideration.. .. We view others as connected as soon as we see a real possibility of activity by either party as bearing on the other, even if no actual activity, let alone interactivity, now connects them or is planned.

(O’Neill 1996, 114)

Applying O’Neill’s account to business, one can say that business ethics involves seeing a client or customer as more than a mere means to an end. So a key presupposition of moral reasoning in business is that one is able to recognize a moral connection with others.

On a polyvalent account of action, we need to show how only one valence of an action reveals a moral connection for the agent without inherently being exclusive of the other meanings or ends integral to the operation of a business, as was the case with MacIntyre’s distinction between internal and external goods. On the polyvalent approach, there are three types of meaning predication basic to speech act theory: locution, illocution, and perlocution. While elsewhere I (Mei 2019a) offer a more a detailed understanding of speech act theory as it applies to work, for the purposes of our focus on business as virtuous practice, we need only note what is entailed at each level of predication:

Locution, or what is the propositional nature of work, has to do with the instrumental end which a *task* of work proposes to achieve. We can say that the propositional statement of work is the task.

Illocution, or the conventional *force* of work, involves meeting standards of excellence when performing a task.

I will come to perlocution in a moment. For now, let us note that both locution and illocution relate to the performance of tasks in view of what MacIntyre would refer to as external goods—e.g. business goals relating to profit, survival, and success. For a summary of the three levels of predication, see Table 1 below.

**Table 1:** Summary of Key Concepts

Speech Act	Meaning Effect	Focus	Illustrative Description
Locution	Instrumental	Task	“To see”
Illocution	Conventional	Force	“To see because …”
Perlocution	Imaginative	Transformation	“To see as …”

At the level of locution, the functioning of a business depends on its many employees to complete tasks involving systems analysis, the operation of machines and technology, sales, customer service, maintenance of real and social capital, and so on. On the critic’s view, the worry is that successful completion of tasks will not connect to a wider sense of ethical consideration when it is appropriate. Heidegger and Marcuse often describe this way of thinking and acting as a technical rationality that is incapable of thinking outside of the means-ends relation. This rationality becomes problematic when tasks have ethical implications. As we know from the COVID-19 pandemic, the task (or locution) of conducting online meetings has ethical implications with respect to impacting negatively on work-life balance or gender equality (Nash and Churchill 2020; Como et al. 2021).

At the level of illocution, there are implicit and explicit references to standards of excellence, or norms of what counts as doing something well and doing something poorly. This can include standards of skill, know-how, and corporate culture. On this view, one might be tempted to think that perhaps illocution might provide a response to MacIntyre’s objection since it may facilitate a recognition of moral relations. It is true that in drawing on norms, illocution might therefore allow for an expansion of one’s moral outlook to the extent that one begins to see relations with others and oneself in a different way. We can think here of how any role having to deal with customer complaints may involve standards of fairness, and how dealing with complaints fairly is not only an end for the business but something edifying for the employee. Fairness might also relate to a broader reflection on fairness as justice in society; or it might link to empathy where, as many in this line of work know, fairness cannot often be achieved without empathy for the customer’s situation and story.

This capacity of illocutionary force should not be discounted. However, it may not be enough by itself since it may not provide the critical force for employees to be able to see that the others with whom they are interacting have a significant moral standing. Customers and clients can disappear when they are seen foremost as items or features within a process or transaction. What is required for the kind of moral recognition with which we are concerned is a critical distance that enables an employee to stand outside what is happening in order to reflect and perceive others in a different way.

Enter Perlocution: Perlocution involves unanticipated effects that outrun the original intention of the task or the object used or produced in work (Mei 2019a, 56–57). These unintended effects involve two key features. First, as unintended they are not necessarily bound to the intentions of the business as expressed by its corporate aims and values. Second, these effects tend to rely heavily on the imagination as a capacity to “see as”. One sees work as something else, in some other relation, and by virtue of that one sees oneself in a different manner. Furthermore, it is often the case that such imaginative acts involve rethinking the relation to others. It should be emphasized here that the imaginative act of “seeing as” is a way of stepping outside the familiar frame of relations in the workplace. It provides the creative and critical viewpoint through which one can question and reflect on what one does in work.

Take for example the idea of role-playing, which is something that has been explored in my consultation work with Quorsus. Quorsus is a high-end financial institutions consultation firm whose analysts are responsible for advice with respect to structural, financial, regulatory, and technological processes and procedures. A single analyst will often have to work with an array of different employees and managers from the financial institution for whom s/he is consulting. So the analyst’s role also requires a great deal of flexibility in adapting to different personalities and styles of management. In short, it is a role that is suited to someone who can deal well with people.

One analyst with whom I was working experiences a great deal of social anxiety and although well qualified to advise on technical matters, she thought that she would struggle in adapting to the social demands of her work. If she can’t work well with her clients, then the technical advice that she has to offer may not be heard well enough or seem as convincing. What she found unexpectedly, however, was that she was able to see herself in a different role—that is, as someone who had to perform in a certain way in order not to let down those who depended on her. She described this responsibility as allowing herself to step outside her social anxiety and, moreover, as something that has allowed her to cope better with social relations in her personal life. On reflection, the analyst realized that she would not have been able to do this by herself and that the supportive culture at Quorsus was essential for her ability to perform her professional role. She identified in particular the culture’s emphasis on openness, or the idea that each employee should make him- or herself available for help and advice. I will return to the importance of openness when discussing virtues in the next section.

For now, let us note that the capacity of the analyst to step outside herself is something that operates at the level of perlocutionary effects. Consider that the analyst’s technical expertise and the quality of advice she provides to her clients involves locutionary and illocutionary ends—namely, to provide answers to what can be improved and to do this well according to technical knowledge of operations and the field of competition. The idea that her work could act as a medium by which she could transform her perception of herself in relation to others is precisely the kind of imaginative exercise which perlocution can disclose. In the case of the analyst, it resulted in the discovery of a new kind of moral solicitude and self-care.

## From Moral Connections to Virtues in Business

Moral awareness is not enough by itself. It requires being practiced. As we saw earlier with the example of temperance, virtues are one way in which moral awareness can be practiced with others. The aim in this section is to show how moral awareness (of the connection with others) can be practiced by virtues when understood through the speech act theoretical account that I have presented. This will involve seeing how virtues promote reflection and action on the moral implications of perlocutionary effects.

Returning to the example of the Quorsus analyst, we can see that what she identified as openness is essentially a virtue that the company takes to be necessary for their employees to develop and for the business to run well. It is perhaps worth underscoring how openness is a virtue by citing what Gabriel Marcel called *disponibilité*, or what is the virtue of being able to make oneself present or available to the other person with the aim of promoting understanding (Treanor 2016). In this sense, we can say that the Quorsus employees engaged in the moral practice of “being present” in order to help the analyst in question better perform her role and develop as a person. There is, of course, much more detail that would require discussion in order to see how a corporate virtue can work well. But from the Quorsus example, we can at least see how practicing a virtue, even within a business setting, provides an opportunity for moral development and reflection.

To be sure, let us take another example from a different kind of business. In my work with a social enterprise cafe called Lily’s Bistro, one of the things I was asked to discuss was how certain virtues were involved in the bistro’s aim of helping people suffering from learning disabilities, mental health issues, criminal pasts, and/or homelessness to become employable. One might think superficially that this merely involves getting them work experience. Partially, this is true. But what is equally important, given the mission of the business, is being able to help the employee develop as a well-rounded person who can work and engage with others. In short, the aim is to make each employee a more capable person.

Our discussion eventually led to how baking a cake involves the virtue of patience—that is, the ability to measure ingredients and time their combination. This virtue is active in the locutionary task of what it is required in baking a cake (i.e. following instructions in a recipe). It is also active in the illocutionary standards of what it means to do so well, such as in knowing how to make the cake flavorful, moist, and appealing in appearance.

The perlocutionary effects arise when the cake enables different kinds of “seeing as”. With respect to the aims of Lily’s Bistro, the task of baking a cake can allow the baker to create a sense of friendship and trust with fellow employees from a shared background. Two employees can struggle with the patience of getting the timing of creaming the butter and sugar, fail on a few attempts, only to succeed finally not just at achieving the right consistency of batter but also in creating a sense of comradery with one another. Moreover, the task and the virtues informing the task can open to wider considerations about the baker herself and the customers who purchase her cake. On the former, the volunteer employment provides work experience and skills that the business owner uses to create pathways for the employees to paid employment with other local businesses. Baking the cake then informs a sense of self-development and self-esteem. On the latter, the customers patronizing the cafe are seen not just as customers but those supporting these pathways. In the end, the cake acts as a focal object enframing relations of care and development beyond simple transactions of production and exchange. In other words, the task of baking a cake and the virtues it enlists activate a moral imagination allowing one to see oneself and others as being non-reducible to relations of commercial exchange and ideally as engendering a greater degree of dignity.

While it is possible in either the case of Quorsus or Lily’s Bistro that the respective advantages and benefits could emerge without a conscious recognition of the specific virtues involved in a task, conscious recognition of the respective virtues provides a framework within which an employee can reflect on her own standing and progress. It widens the sense of perspective so important in enabling a person to expand her sense of what is possible in terms of a flourishing life. Moreover, we should not forget that for the business itself, a conscious recognition and development of business-specific virtues enable that business to commit to making its work more meaningful for its employees. To speak of profit and success, on the one hand, and employee personal and moral development, on the other, should not be mutually exclusive. Indeed, on the speech act theoretical account that I have presented, the goods of business excellence and of moral excellence can amicably coincide. We can perhaps think of the business as providing a community that offers the time and space for moral practice as well as a source of livelihood and success.

## A Response to MacIntyre’s Objection

To recall, the objection to taking business as an example of virtuous practice is founded on a general tension between the internal and external goods distinction that lies at the heart of MacIntyre’s account of virtue ethics. While the two goods themselves are not necessarily mutually exclusive, MacIntyre emphasizes that when virtues are set within a context governed by the aims of business, they become so. The aims of profit, success, etc. will compromise and potentially corrupt whatever virtues may be employed. I referred to this as an error in the ordering of the goods.

While the polyvalent approach allows both the external goods of business (locutionary and illocutionary ends) to exist alongside the internal goods of moral agency (perlocutionary effects), the worry is that this amicability loses any critical force that would allow an employee to recognize when compromise or corruption of the virtues emerges—that is, when things become morally mis-ordered. It is Sinnicks’ allegation that businesses simply lack the critical capacity and willingness to do so. Thus, external regulation presents the most compelling means of assuring businesses to act ethically.

The polyvalent approach seems to be a bit naïve in the face of this account. It assumes that a business will in fact want to take matters of morality and employee well-being seriously. While I acknowledge that any assumption about what a business will and will not take seriously is significant, the approach that I am forwarding is actually not guilty of making such assumptions. Rather, it is the approach of MacIntyre and Sinnicks that assumes businesses are incapable of exercising some form of moral reasoning. Consider that on MacIntyre’s account business is by default an organization that is concerned only with external goods. If one looks to the past or present, there are far too many examples to support this assumption—most of which orbit major recessions or involve prioritizing the responsibility to stockholders as opposed to consumers. However, this way of defining business relies on historical examples. The examples themselves say nothing definitive about business. Can’t business be characterized differently and plausibly so? If one can do so, might it have an impact on how businesses think and perceive of themselves such that they might see themselves as having some degree of moral responsibility?

Far from assuming businesses have a certain nature, I am operating under the idea that how businesses act is largely a function of the expectations that underwrite their conceptual environment of operation. Change these environmental conditions, and you can change how businesses operate. But, of course, this still leaves Sinnick’s view on the table. Why not regulate as a way of changing these conditions?

As I argued earlier, regulation only results in a limited form of responsibility whose object is compliance with specific regulations. Any action or consequence not covered by these regulations is in theory permissible. What one would want instead is for businesses to have a richer sense of moral responsibility, where they are self-regulating because they care about their standing as a moral agent (assuming businesses can be characterized as agents). In other words, the change in the conceptual environment for business operations is that we expect they are the kinds of entities that are capable of moral reasoning and responsibility.

Granting this capacity comes with an increased demand and expectation for responsibility. To be sure, this is a classic feature of accountability for the virtue ethicist—i.e. the capable moral agent will be able to take responsibility for her agency by providing some justificatory account of why she committed to performing an action. Robert Solomon’s (1992a, 325; b, 145–152) account of businesses as kinds of communities is that which comes closest to this approach. But there is also an amicable link to MacIntyre (against his own intentions). One can say that communities are capable of the kind of critical reasoning that allows them to revisit and revise their values, aims, and principles. MacIntyre (1988) notes one major cause of revision is the pressure from other communities (or rival traditions, as he puts it) that present competing examples and reasons.

So with business, where does this pressure come from if not some external authority? It must come from within the sphere of business itself—that is, from those who are potential clients and customers as well as from a business’s competitors. If the expectations of the client and customers change and expect moral accountability, and if competitors find meeting these expectations is effective and desirable, then the conditions for doing business will have begun to change.

There is one last feature determining the conditions for business operation that cannot be forgotten. Even if perlocutionary effects allow an employee to engage and develop a capacity for self-critical reflection about her relations to herself and others, MacIntyre’s other worry is that even if this capacity is present, it may be severely diminished or incapacitated by the business environment. So it is crucial that if there is a significant change in the conditions and expectations of what businesses are responsible for, then businesses must take seriously the aim of developing structures and processes to support their employees’ capacity for self-critical reflection. We saw illustrative examples of this in how Quorsus and Lily’s Bistro aimed to discern what virtues were involved in their respective employees’ tasks and how this could be thematized and practiced in various ways. In short, the business culture was about caring about the business as well as its people. That is to say, the workplace is more than just an opportunity to earn a wage or turn a profit; it is place in which one can be a member of another kind of moral community.

If the foregoing response to MacIntyre’s objection is sufficient, then it would seem that in practice the speech act approach to business virtues still has one potential weakness. It requires a lot of things to come into place—e.g., a supportive culture, working out what virtues are involved in a business, working out how to specifically support employees, etc. The speech act approach is effort and resource intensive. But isn’t this effort and demand on resources what we expect to be involved in striving to meet the high standards of excellence in the moral sphere of action?

## Conclusion: What Practical Changes in View?

In my concluding remarks, I want to address the foregoing concern about effort and resource. In what follows, I offer some practical steps that might help facilitate the process of transformation by noting how businesses can start thinking about strategies for incorporating virtues.

If virtues are essential to the execution of the locution and illocution of tasks as well as the development of and reflection on the imaginative moral connections arising from the perlocution of tasks, then discovering what virtues matter to any particular business is simply another way that a business can better understand itself and its employees. Businesses, especially during transition and growth, often have to revisit what kind of organization they are and how they function. So the work of discovering appropriate virtues is only a further development of this process.

Call this *the onus of self-discovery*, where one attempts to see what a business requires in order to flourish in multiple ways (per the speech act sense of polyvalence). One proven method to help this process of self-discovery is a phenomenological approach to work (Madsbjerg 2017, 15–18) in which managers and officers aim to gain a better sense of the experience of the employees and customers in relation to business tasks. Interviews, focus groups, and action learning sets may be tools to help disclose this sense from the employee or customer point of view; but additionally, it is also important for officers and managers to gain experience or renew their experience of the everyday tasks themselves by having to do them.

The process of self-discovery can then begin the task of identifying what virtues matter to a business. Call this *particularism* about how we analyze virtues in business.^5^ That is to say, one gives an account of how bespoke virtues operate within contexts specific to and respective of a business and its aims and values. Each virtue can only be made sense in relation to tasks within a specific business culture (cf. Solomon 2003). So thinking about the virtues means there is no objective list of the virtues that will work for each business.

Finally, if making moral connections relies on an imaginative capacity, then businesses will benefit by cultivating the imagination more generally. There are many methods already used by businesses—such as, team-building exercises, rotational work, changing the work environment, external facilitation of critical discussion of work issues, and so on—that can be redesigned or reinvigorated to help cultivate a greater capacity for imagining and building on the ideas the imagination discovers. Of course, the importance of the imagination in the business world has not gone unheralded by academics when demonstrating how employees benefit from being involved in what I have called the perlocutionary dimension of work (e.g. Moberg 2000, 851–853; Madsbjerg 2017, 125–165). In practice, nonetheless, businesses need to think of themselves as offering imaginative practices as an opportunity for their employees to discover more about themselves and their relations with others.

And why not? If the workplace is that in which we spend the majority of our time, should we not strive to make it another kind of community for our development and flourishing? Surely, work can make us better persons?

## Footnotes

1 While happiness (*eudaimonia*) is the end or telos of human activity as such, the reason why acting virtuously is done for its own sake is because it is considered an enacting or practicing of happiness. In other words, we aim for happiness as an end, yet we achieve it as an activity or active practicing. See Mei (2019b).

2 MacIntyre (2016, 170–172) does recognize that advanced economies can enlist practices in view of common social goods. But this is more the exception.

3 Beadle and Knight (2012) make a persuasive case for showing how virtues, practices, and internal goods can have a positive effect in view of meaningful work. However, an uneasy tension remains insofar as their position seems to settle on an antagonism between what a worker might find meaningful and what a business takes to be so.

4 For an account of how Ricoeur draws on J. L. Austin and John Searle in order to develop his own theory, see Mei (2019a).

5 The particularist approach differs from other theories about virtues in business that tend to draw virtues from other traditions and/or create a core list of virtues for business (e.g. Alzola 2012; Arjoon 2000; Audi 2012; Bertland 2009; Bright et al. 2014; Crossan et al. 2013; Ewin 1995; Fontrodona et al. 2013; Grant et al. 2018; Kirchengast 2011; McGhee and Grant 2018, pp. 481–484; Moberg 1999; Moore 2005, pp. 655, 676–677, Moore 2008, Moore 2015, Moore 2017; Newton 1992, pp. 364–365; Schudt 2000; Shanahan and Michael 2003; Solomon 1992a, 1992b, 1999, 2003; Vriens et al. 2018; Wang et al. 2016; Whetstone 2001, p. 111) or the market (Maitland 1997). Risks of this approach include: assuming businesses are similar enough for core virtues to apply; importing virtues from a particular culture or religion without critical attention to matters relating to race, gender, etc.; confusing what a virtue from one culture means and how it might apply or even conflict with the business.
